# News and Notes

**Published:** 1900-10

**Authors:** 


### *NEWS AND NOTES.*

---

DR. T. V. HUBBARD has removed his offices from the Fitten building to the Austell.

---

DR. CLAUDE SMITH has returned from Philadelphia and Baltimore, where he has been for the last two months doing special work in pathology and bacteriology.

---

DR. HUNTER MCGUIRE died in Richmond, Va., on September 19. He had a stroke of paralysis early in the summer, from which he never recovered. Dr. McGuire was one of the most distinguished surgeons in the South.

---

DR. MICHAEL HOKE, who has been studying in Boston at the Massachusetts General Hospital for the last four months, has returned to Atlanta. Dr. Hoke has been working with Dr. Bradford, doing especially orthopedic work.

---

THE Southern Surgical and Gynecological Association will be held in Atlanta on November 13, 14, 15. Dr. A. M. Cartledge, of Louisville, is thc president and Dr. W. E. B. Davis, of Birmingham, is secretary. A prosperous meeting is expected.

---

THE gastric juice of the dog is now on sale at the St. Petersburg pharmacies. It comes from the Institute of Experimental Medicine, of that city, and is said to be far more efficacious as a digestive ferment than all other preparations on the market.

---

THE meeting of the Southern Surgical and Gynecological Association will be held in Atlanta, November 13th, 14th and 15th, under the presidency of Dr. A. M. Cartledge, of Louisville. Prospects are splendid for successful session. Members of the medical profession are cordially invited to attend.

---

DR. HENRY F. HARRIS, who for the last four years has been assistant professor of pathology in the Jefferson Medical College, Philadelphia, has accepted the chair of professor of pathology and bacteriology in the Atlanta College of Physicians and Surgeons. The many friends of Dr. Harris welcome him home again.

---

ARTHUR GOEBEL, brother of the dead governor and his devisee under the will, has placed a claim against the estate of Governor Goebel for $10,000 for Dr. McCormack's services. Governor Goebel and Dr. McCormack had long been close friends, and when Goebel was shot Dr. McCormack, who happened to be in Frankfort at the time, was one of the first physicians to come to his assistance. The wound was mortal, but knowing that if life could be prolonged a few days the legislature would decide the contest, and that in the state of feeling resulting from the assassination Goebel would probably be declared governor, Dr. McCormack took every step known to science to maintain life. Dr. McCormack refused to render any account for his services on the ground of personal friendship.

---

DR. C. A. DAVIES of the Isle of Man in a recent communication to the British Medical Association said: Consanguinity in marriage among the Manx people had led to much illness in the offspring. There has been little crossing of races among them since the twelfth century, and a custom exists on the island to-day of discouraging marriages between persons living in different parishes. Hence there exists all over the island a condition of close "inbreeding." As a consequence the general death-rate from consumption is 25.70 per 10,000, double that of England and Wales. In Lonan, an isolated part, where all of the inhabitants of the parish have only three or four surnames, the rate is 41.79 per 10,000, while in Peel, where many more strangers come, it is only 15.19.

---

THE London *Chronicle* states that Dr. Otto Emmerisch of Baden-Baden has discovered an alleged specific for morphinism. It is said to be a distilled vegetable oil of intense acidity, two or three drops of which are taken internally daily. It produces an intense loathing for morphia, after three to six weeks of its use. Dr. Robert Koch reports from German New Guinea that he has been able to protect himself and colleagues from malaria and believes his investigations will greatly assist in combating the disease. We also learn that members of the African expedition of the Liverpool School of Tropical Medicine have found the cause of elephantiasis; they state that it is propagated in the same way as malaria. Reports from all these sources are fragmentary. We shall await with interest more specific information.

---

AT a meeting of nurses who served during the Spanish-American war, held at the New York Hospital on Thursday, September 6th, some sixty nurses being present, a constitution was adopted, and those nurses only were declared eligible who had served not less than one month. This limitation makes about five hundred nurses eligible for membership. The list of officers is as follows: President, Dr. Anita Newcomb McGee, acting assistant surgeon, United States army, detailed to the army nurse corps, now with the surgeon general's office, Washington; vice presidents, Dr. L. A. Hughes, Boston; Miss Hibbard, Manchester, N. H.; Dr. Isabel Cowan, detailed to duty at the Presido Reservation, California; Miss Hobson, New York; Miss McCloud, now serving in the

Philippines; Miss Walton, New York; Miss Miselback, now serving in Santiago, Cuba; Miss Robbins, Miss Dreyer, now in the Philippines; Miss Read, Baltimore; recording secretary, Miss Lela Wilson, Boston; corresponding secretary and treasurer, Mrs. George Lounsbury, Charleston, S. C. The association will meet in Washington next year.

---

NEW HOME FOR J. B. LIPPINCOTT COMPANY.—An important transaction has just been concluded by which a number of old-fashioned dwelling houses on East Washington Square have passed from the ownership of the heirs of the famous lawyer, Horace Binney, and will soon be torn down to make way for a fine building to be occupied by J. B. Lippincott Company, whose old home on Filbert street, above Seventh, was burned down some months ago. Possession is to be given by September 14, and it is expected that the demolition of the old structures will begin soon after. The site is considered a very eligible one for the Lippincott Company, as it has light on three sides, is very central, and they will be enabled to promptly issue and increase their excellent line of medical publications by standard authorities. By the way, their new catalogue, just issued, is handsomely illustrated with excellent portraits of many of America's leading medical writers.

Many historic recollections cluster about the properties just sold. They stand on the ground once occupied by the old Walnut Street Prison, built before the Revolution, and in which during the struggle the English confined American prisoners during the former's occupation of Philadelphia.

---

***NOTICE.—This JOURNAL and THE INTERNATIONAL JOURNAL OF SURGERY one year for $1.00 cash, to new subscribers. No out-of-town checks accepted unless 10c. is added for cost of cashing. Strictly to new subscribers.***

### WHEN PAPA'S SICK.

When papa's sick, my goodness sakes!
Such awful, awful times it makes.
He speaks in G, such lonesome tones,
And gives such ghas'ly kind of groans,
And rolls his eyes, and holds his head,
And makes ma help him up to bed;
While Sis and Bridget run to heat
Hot-water bags to warm his feet,
And I must get the doctor quick—
We have to jump when papa's sick.

When papa's sick ma has to stand
Right 'side the bed and hold his hand;
While Sis, she has to fan and fan,
For, he says, he's a "dyin' man,"
And wants the children round him, to
Be there when "suffering pa gets through."
He says he wants to say good-bye
And kiss us all, and then he'll die;
Then moans, and says his "breathin's thick"—
It's awful sad when papa's sick.

When papa's sick he acts that way
Until he hears the doctor say:
"You've only got a cold, you know;
You'll be all right in a day or so."
And then—well, say, you ought to see—
He's different as he can be,
And growls and swears from noon to night
Just 'cause his dinner ain't cooked right,
And all he does is fuss and kick—
We're all used up when papa's sick.—Ex.

---

THE following new books are announced by W. B. Saunders & Co., Philadelphia:

Illustrated Medical Dictionary, indexed by W. A. N. Dorland. Price, $4.50 net, $5.00 net.

"Modern Medicine," by Drs. J. L. Salinger and F. J. Kalteyer, of Jefferson Medical College, Philadelphia. Price, $4.00 net.

"Rhinology, Laryngology and Otology, and their significance in General Medicine," by Dr. E. P. Friedrich, of the University of Leipsig, and Dr. H. Holbrook Curtis, of New York. Price, $2.50 net.

"A Text Book of Histology," by Drs. Bohm and Davidoff, of Munich, and Dr. G. Carl Huber, of Ann Arbor, Michigan. Ready in October.

"Essentials of Histology," by Dr. Louis Leroy, of Vanderbilt University. Price, $1.00 net.

"Surgical Technic for Nurses," by Emily A. M. Stoney, author of "Stoney's Nursing."

NEW EDITIONS.

"Anders' Practice of Medicine," 4th edition. Price, $5.50 net.

"McFarland's Bacteriology," 3rd edition, revised and enlarged. Price, $3.25 net.

"Hyde & Montgomery's Venereal Diseases," new enlarged edition. Price, $4.00 net.

"American Text-Book of Physiology," 2d edition revised, in two volumes. Volumn 1 now ready. Price, $4.00 net per volume.

"Saunder's Pocket Formulary," 6th edition increased in size by over 200 formulæ. Price, $2.00 net.

"Garrigues' Diseases of Women," 3rd edition. Price, $4.50 net.

"DaCosta's Surgery," 3rd greatly enlarged edition. Price, $5.00 net.

"Stengel's Pathology," 3rd edition revised. Price, $5.00 net.

During the next six months we shall issue from four to six volumes in Saunders' Medical Hand-Atlas Series.

This firm also announces the establishment of a London branch.

